# The early landscape of coronavirus disease 2019 vaccine development in the UK and rest of the world

**DOI:** 10.1111/imm.13222

**Published:** 2020-07-08

**Authors:** Hannah R. Sharpe, Ciaran Gilbride, Elizabeth Allen, Sandra Belij‐Rammerstorfer, Cameron Bissett, Katie Ewer, Teresa Lambe

**Affiliations:** ^1^ The Jenner Institute Nuffield Department of Medicine University of Oxford Oxford UK

**Keywords:** coronavirus disease 2019, severe acute respiratory syndrome coronavirus‐2, vaccine

## Abstract

Since the first World Health Organization notification on 31 December 2019, coronavirus disease 2019 (COVID‐19), the respiratory disease caused by the coronavirus severe acute respiratory syndrome coronavirus‐2 (SARS‐CoV‐2), has been responsible for over four million confirmed infections and almost 300 000 deaths worldwide. The pandemic has led to over half of the world's population living under lockdown conditions. To allow normal life to resume, public health interventions will be needed to prevent further waves of infections as lockdown measures are lifted. As one of the most effective countermeasures against infectious diseases, an efficacious vaccine is considered crucial to containing the COVID‐19 pandemic. Following the publication of the genome sequence of SARS‐CoV‐2, vaccine development has accelerated at an unprecedented pace across the world. Here we review the different platforms employed to develop vaccines, the standard timelines of development and how they can be condensed in a pandemic situation. We focus on vaccine development in the UK and vaccines that have entered clinical trials around the world.

## Graphical abstract

The SARS‐CoV‐2 pandemic has accelerated vaccine development at an unprecedented pace across the world, whilst utilising novel platform technologies. This review summarises the main platforms employed for vaccine development, the standard timelines of development and how they can be condensed during a pandemic. We focus on vaccine candidate development in the UK, and vaccines which have progressed to phase I clinical trials in the UK and worldwide.

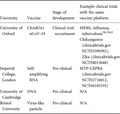

**Abbreviations**
ADE: antibody‐dependent enhancement
APC: antigen‐presenting cell
CEPI: Coalition for Epidemic Preparedness and Innovations
ChAd: chimpanzee adenovirus
COVID‐19: coronavirus disease 2019
DC: dendritic cell
DRC: Democratic Republic of the Congo
EBOV: Ebola virus
GMP: good manufacturing practice
hAd: human adenovirus
IL: interleukin
LAV: live attenuated vaccine
MERS: Middle Eastern respiratory syndrome
S: Spike glycoprotein
SARS: severe acute respiratory syndrome
Th2: T helper type 2
VLP: virus‐like particle
VSV: vesicular stomatitis virus

## Introduction

December 2019 saw the outbreak of a novel beta‐coronavirus from Wuhan, China. This virus, severe acute respiratory syndrome coronavirus 2 (SARS‐CoV‐2), and the corresponding disease – coronavirus disease 2019 (COVID‐19) – causes illness ranging from asymptomatic to severe respiratory distress, pneumonia and death.^1^ Although its exact origins are unknown, SARS‐CoV‐2 shares genetic homology with other coronaviruses found in bats and with its most closely related human virus, SARS‐CoV‐1.^2^, ^3^ Beta‐coronaviruses are the causative agents of two previous outbreaks this millennium. The SARS‐CoV‐1 outbreak in 2003 caused similar respiratory pathology and amassed 8098 cases and 774 deaths.^4^, ^5^ SARS‐CoV‐1 potentially underwent zoonotic transmission between bats and palm civets before human infection.^6^ Middle Eastern respiratory syndrome coronavirus (MERS‐CoV) is another related CoV identified in Saudi Arabia in 2012. Consequent outbreaks have determined a mortality rate of approximately 35%, although this does not include likely asymptomatic transmission.^7^, ^8^ Similarly, genomic evidence suggests that MERS‐CoV circulated between bats and camels before transmission to humans,^9^, ^10^ suggesting that bats are a natural reservoir species of coronaviruses, and that zoonosis can occur with close human‐to‐animal contact.^8^ Two other beta‐coronaviruses, HCoV‐OC43 and HCoV‐HKU1, and two alpha‐coronaviruses, HCoV‐229E and HCoV‐NL63, circulate within the human population and cause mild respiratory pathogenesis.^8^

COVID‐19 mortality is currently estimated to be approximately 3·4% globally,^11^ with a higher risk of COVID‐19 mortality in older adults and those with pre‐existing co‐morbidities.^12^, ^13^ Since January 2020, SARS‐CoV‐2 has spread to more than 200 countries and caused over four million infections and over 300 000 deaths.^14^ COVID‐19 was declared a global pandemic by the World Health Organization on 11 March 2020.^15^

This outbreak of a previously unknown virus has instigated a worldwide effort to develop a vaccine for COVID‐19. The traditional vaccine development pipeline, which takes an average of 10 years from conception to licensing,^16^ is unfeasible for a situation of this urgency. Therefore, new strategies for the accelerated development of vaccines, subsequent pre‐clinical and clinical trials, and rapid upscaling of manufacture are necessary to ensure there is no setback in the delivery of potential vaccine candidates. Currently, it is estimated that a vaccine for COVID‐19 will be widely available in 18–24 months if clinical trials demonstrate safety, immunogenicity and protective efficacy.^17^

## Vaccination against COVID‐19

Vaccination can generate long‐lasting immunity by exposing individuals to antigens, to drive the development of immunological memory before encounter with live pathogen. The resulting immunity can be mediated by humoral antibody induction, and/or cellular T‐cell effector function.

Spike glycoprotein (S) is the sole surface protein of the SARS‐CoV‐2 virion.^18^ SARS‐CoV‐2 S mediates viral entry into host cells via angiotensin‐converting enzyme 2, which is expressed at high levels on the surface of pulmonary epithelial cells.^19^

Currently, the most clinically advanced COVID‐19 vaccines target the S. In addition, the structure of the SARS‐CoV‐2 S shares homology with SARS‐CoV‐1 S, the primary target of the immune response and vaccine development for SARS.^20^, ^21^ The correlates of protection against SARS‐CoV‐2 are not yet fully understood, so it may be necessary that vaccination delivers robust humoral and cellular immunogenicity to increase the likelihood of inducing protection.

Humoral protection arises through structural epitope recognition of proteins. In SARS‐CoV‐2 infection, antibodies are most frequently generated against the S protein and the internal nucleoprotein.^22^ Potential SARS‐CoV‐2 vaccines would ideally generate a long‐lasting humoral immunity with protective titres of neutralizing antibodies that do not cause antibody‐dependent enhancement (ADE) upon re‐infection. ADE occurs when antibodies to the pathogen, or cross‐reactive antibodies to a closely related pathogen, facilitate viral infection of cells instead of protecting the host and contribute towards an exacerbated pathology.^23^ ADE was detrimental in SARS‐CoV‐1 pathogenesis during the 2002/03 outbreak,^23^ and pre‐clinical trials demonstrated infection of macrophages via ADE,^24^, ^25^ and lethal pneumonia in mice.^26^, ^27^ ADE is therefore of potential concern for a SARS‐CoV‐2 vaccine.

Cellular immunogenicity is vital for vaccine‐derived immunity against intracellular pathogens, and for a rapid cytotoxic response to re‐infection.^28^, ^29^ However, acute SARS‐CoV‐2 infection may be worsened by a skewed immune response towards CD4^+^ T helper type 2 (Th2) cells involving interleukins 10 and 14 (IL‐10 and IL‐14), which initiates immunosuppression of inflammatory CD8^+^ T‐cell responses and could contribute towards more severe pathology.^30^, ^31^ Therefore, vaccines that do not induce a skewed Th2 immune response are thought to be optimal for SARS‐CoV‐2.

An adjuvant is a substance that is often co‐administered with a vaccine to enhance the immunogenicity and duration of protection by stimulating the innate immune system. The choice of adjuvant can also skew the T‐cell response towards a Th1‐ or Th2‐dominant reaction, necessitating careful planning when trying to avoid adverse events such as ADE.^32^

## Platforms for COVID‐19 vaccine development

### Whole virion vaccines (live attenuated and inactivated)

Live attenuated vaccines (LAV) are viruses that are rendered replication‐incompetent through repeated passage in cell culture, and inactivated vaccines use whole pathogen that has typically been killed by exposure to chemicals (e.g. formaldehyde) or heat inactivation.^33^ LAV are immunogenic and reproduce the breadth of the humoral and cellular immune protection that would be generated by live viral infection;^32^, ^33^ however, inactivated vaccines are generally less immunogenic and require more than one dose or an additional adjuvant.^34^

Safety issues regarding the generation and subsequent attenuation of the virus, with potential for re‐activation in vaccinated individuals, means LAV are not a tenable vaccine strategy for highly pathogenic viruses.^33^, ^35^ This also prevents immunization of individuals with weakened immune systems who are at further risk of illness if the pathogen reverts.^36^ From the perspective of vaccine distribution, LAV are generally kept refrigerated to preserve immunogenicity, which may be problematic in countries that cannot sustain cold‐chain distribution.^34^, ^36^

Live attenuated vaccines for SARS‐CoV‐1 were tested in pre‐clinical trials.^37^ There is currently one company, Codagenix (Farmingdale, NY), proposing a computationally designed, lab‐made SARS‐CoV‐2 ‘virus’ that is immunogenic but not pathogenic.^38^ SinoVac (Beijing, China) demonstrated safety and immunogenicity of an inactivated SARS‐CoV‐1 vaccine in a phase I trial,^39^ and have determined efficacy of a formalin‐inactivated SARS‐CoV‐2 vaccine in rhesus macaques^40^ (Table 1). Although this vaccine did not demonstrate any ADE‐derived pathogenesis, previous whole virus SARS‐CoV‐1 vaccines trialled in mice induced eosinophil‐derived immunopathology upon viral challenge,^27^ and Th2‐driven histopathological changes in the lungs.^31^

**Table 1:** Categories of vaccine, with licensed and experimental examples

Vaccine	Licensed vaccines	Example institutions developing COVID‐19 vaccines
Live attenuated	Smallpox, measles, yellow fever, mumps, influenza, rubella, varicella, polio	Codagenix (Farmingdale, NY)
Inactivated	Inactivated polio, rabies, hepatitis A	SinoVac (Beijing, China)
Protein subunit	Hepatitis B, *Haemophilus influenzae* B, pertussis, human papillomavirus, pneumococcal bacteria, meningococcal bacteria	Generex Biotechnology (Miramar, FL) Vaxart (San Francisco, CA) Medicago (Quebec City, Canada) GSK (London, UK) and Clover Biopharmaceuticals (Chengdu, China) University of Queensland
Nucleic acid	None in humans	Moderna (Cambridge, MA) Inovio Pharmaceuticals (Plymouth Meeting, PA) Pfizer (New York, NY) Imperial College London Takis Biotech (Rome, Italy) BIOCAD (St Petersburg, Russia)
Viral‐vectored	Japanese encephalitis (Australia), dengue virus (Mexico and Brazil)	University of Oxford CanSino Biologics (Tianjin, China) Greffex (Houston, TX) Janssen (Beerse, Belgium)

For each category of vaccine listed, the table provides examples of licensed vaccines and institutions developing experimental COVID‐19 vaccines.^70^, ^89^

### Protein subunit vaccines

Protein subunit vaccines include antigenic proteins thought to induce a protective immune response. This vaccine type is produced *in vitro* and circumvents handling highly pathogenic live viruses.^33^, ^41^ Subunit vaccines predominantly elicit a humoral antibody response, and most are administered with an adjuvant, which is a prerequisite to stimulate a strong immune response and generate a higher‐quality immune memory in humoral and cellular compartments. However, the inclusion of adjuvants can increase the reactogenicity and production costs, which are important considerations.^41^ Virus‐like particles (VLP) are a type of subunit vaccine that present many copies of the relevant antigen in a three‐dimensional virus‐like structure, and may be immunogenic enough to not require the inclusion of adjuvants.^41^

Subunit vaccines are an attractive vaccine technology for rapid vaccine development, and multiple institutions worldwide are developing protein subunit‐based vaccines (Table 1). They can be upscaled for mass production at good manufacturing practice (GMP) standards,^42^ and distribution has less reliance on cold‐chain systems.^34^ However, they can require bespoke manufacturing processes, which can increase cost, and may require specific mammalian cell expression and optimization.^18^, ^43^

### Nucleic acid vaccines

Similar to subunit vaccines, specific proteins from the target pathogen are chosen for their immunogenic epitopes; however, these proteins are delivered as either plasmid DNA or RNA sequences.^44^, ^45^ Upon vaccination, the host cell manufactures the pathogen protein, which is recognized by the immune system as foreign and generates an immune response.^44^ Non‐capsulated RNA vaccines are readily removed by the host cell upon injection, so advances in delivery technology, including encapsulation of RNA in liposomes, have been developed to avoid degradation.^46^

RNA vaccines have been shown to induce antigen‐specific antibody and polyfunctional T‐cell responses in phase I clinical trials of cancer vaccines,^46^ and functional antibodies against rabies virus glycoprotein;^47^ however, there are currently no licensed RNA vaccines for humans. Although DNA vaccines are immunogenic in small animal models, they show less immunogenicity in human clinical trials and require adjuvants or multiple doses.^34^, ^48^ Four DNA vaccines are available for animal use;^46^ however, there are currently none licensed for humans.^49^

There are several nucleic acid vaccines in development for COVID‐19 prophylaxis (Table 1). Nucleic acid vaccines are relatively cheap and rapid to manufacture, with the possibility to mass‐produce large‐scale GMP product.^50^

### Recombinant viral‐vectored vaccines

Recombinant viral‐vectored vaccines use the host’s innate immunity to generate self‐adjuvanted immunogenicity, while eliciting a targeted immune response against genetically encoded pathogen antigens.^51^ The viral vector ‘backbone’ is constructed from a genetically modified virus,^52^ examples being adenoviruses, poxviruses and vesicular stomatitis virus (VSV).^52^, ^53^ This vector typically has insertion sites for gene(s) of the target pathogen, which are expressed intracellularly in the host upon vaccination.^54^

Important considerations for development of virus vectored vaccines is the generation of immunity towards the vector, which could hinder the antigen‐specific response upon a boost vaccination. However, reports from pre‐clinical and clinical studies show sufficient protection can be elicited from a single dose.^55^, ^56^

Human adenoviruses (hAds) are a frequently used viral vector thaat circulate at high frequency in most populations,^57^ contributing towards demographically variable yet significant pre‐existing immunity that can reduce vaccine efficacy.^54^ Vectors constructed from chimpanzee adenovirus (ChAd) were developed to elicit immunogenicity that is similar or superior to that of hAd vectors, while having significantly reduced seroprevalence and hence neutralizing antibodies in most populations.^28^, ^58^ In pre‐clinical studies, ChAd vectors have demonstrated up to 100% efficacy with a single vaccination against several emerging pathogens.^56^, ^59^ Clinical trials have established that ChAd vectors also have a good safety profile and immunogenicity for influenza A virus,^60^ Ebola virus (EBOV),^61^ and MERS‐CoV.^62^

Adenovirus vectors can be rapidly made to GMP at large scale, and a single vaccination can be sufficient to provide rapid immunity in individuals.^63^ This rapid production and distribution pipeline was tested during the 2013–2016 EBOV outbreak in Guinea, Liberia and Sierra Leone, where five viral‐vectored vaccines were rapidly escalated to clinical trials.^63^ A recombinant VSV vector expressing the EBOV glycoprotein (rVSV‐ZEBOV) progressed to phase III trials in Guinea and Sierra Leone and provided 100% efficacy across 4359 individuals vaccinated with a single dose.^55^ Following the second EBOV outbreak in the Democratic Republic of the Congo (DRC) in 2018, the World Health Organization allowed compassionate use of rVSV‐ZEBOV in the DRC; and it has now been licensed in the DRC, Burundi, Ghana and Zambia.^64^ An Ad26‐vectored EBOV vaccine has also been developed by Janssen (Beerse, Belgium) and tested extensively in a prime‐boost regimen in sub‐Saharan Africa for efficacy and immunogenicity.^65^

### Timelines in vaccine development

Development of vaccines is a long process, taking at least 10 years per vaccine.^66^ Most of the duration of vaccine development is determined by clinical trials, which are split into three phases between pre‐clinical exploratory work and licensure of the vaccine:^67^

- Phase I: First‐in‐human experiments on a small number of healthy volunteers, who have not been exposed to the pathogen. The trial focuses on safety and immunogenicity of the vaccine.^67^
- Phase II: Vaccines successful in phase I move into phase II trials. Phase II trials have a greater focus on the immunogenicity of the vaccine and expand the cohort across a wider breadth of the population, allowing for immune response to be analysed across age, gender, ethnicity and other variables.^67^ Efficacy may also be assessed at this stage, with controlled human microbial infection studies giving a useful early indication of potential efficacy for diseases where robust controlled human microbial infection studies models are available.^68^
- Phase III: the efficacy of the vaccine is assessed across a larger population. Phase III studies enrol enough participants to ensure statistical power to assess if the immune response stimulated by the vaccine is sufficient to protect against disease. The clinical end point of phase III vaccine studies is often determined by reduction in case numbers or severity of disease in the cohort and requires an active outbreak.^67^

### Phase overlap

Vaccine trials are extremely expensive,^17^ representing a huge financial risk, and as such the vaccine timeline is extensive.^66^ It is estimated to cost US$31–68 million to bring a candidate to the end of phase IIa trials.^69^ Ethical acceleration of the trial can be achieved by performing phase I/II and phase II/III studies in parallel once sufficient data have been extracted from the preceding phase.^70^ This can entail a larger risk from commercial investors and therefore requires philanthropic organizations such as the Coalition for Epidemic Preparedness Innovations (CEPI) and others to fund the parallel phases to accelerate vaccine development through clinical trials at a rapid rate.^70^

## COVID‐19 vaccine development: the UK perspective

There are a number of COVID‐19 vaccines under clinical development at research institutes in the UK, at least three of which are at the pre‐clinical stage, and one of which has progressed to clinical trial recruitment (Table 2).

**Table 2:** COVID‐19 vaccine development in the UK

University	Vaccine	Stage of development	Example clinical trials with the same vaccine platform
University of Oxford	ChAdOx1 nCoV‐19	Clinical trial recruitment	MERS, influenza, tuberculosis^28^, ^29^, ^62^ Chikungunya (clinicaltrials.gov NCT03590392), Zika (clinicaltrials.gov NCT04015648)
Imperial College, London	Self‐amplifying RNA	Pre‐clinical	MTP‐CEPBA (clinicaltrials.gov NCT02716012, NCT04105335)
University of Cambridge	DNA	Pre‐clinical	N/A
Bristol University	Virus‐like particle	Pre‐clinical	N/A

This table outlines UK universities developing COVID‐19 vaccines, the vaccine platform under development, the stage of development for each vaccine, and examples of previous or ongoing clinical trials for this vaccine platform co‐ordinated by the university.

### Pre‐clinical COVID‐19 vaccine research

A self‐amplifying RNA vaccine encoding SARS‐CoV‐2‐S is under development at Imperial College, London. Previous self‐amplifying RNA vaccines against influenza A virus haemagglutinin and *Toxoplasma gondii* NTPase‐II have induced cellular and humoral immunogenicity in mice,^71^, ^72^ and a first‐in‐class clinical trial using self‐amplifying RNA against hepatocellular carcinoma encoding the transcription factor C/EBP‐*α* demonstrated acceptable safety and tolerability.^73^ This COVID‐19 vaccine is currently in pre‐clinical testing, with a target clinical trial date set for Summer 2020.^74^

Bristol University, with spin‐out company Imophoron (Bristol, UK), is developing a SARS‐CoV‐2‐S VLP vaccine with their adenovirus‐based ADDomer© technology. Imophoron states that the ADDomer© VLP platform has many of the main benefits of VLP‐based vaccines, including rapid development, self‐adjuvant properties and no cold storage requirement.^75^ An ADDomer© VLP vaccine expressing Chikungunya virus E2 protein also proved immunogenic in pre‐clinical trials,^76^ although this platform has not yet been tested in humans.

The University of Cambridge and DioSynVax (Cambridge, UK) are using a bioinformatic approach to design an optimal SARS‐CoV‐2 genetic sequence, which will be used to make a vaccine encoding SARS‐CoV‐2‐S.^77^

### UK COVID‐19 vaccine clinical trials

The University of Oxford COVID‐19 vaccine has recently entered clinical trials in the UK (Table 2). This approach, using viral‐vectored vaccine technology, is being led by the Jenner Institute and the Oxford Vaccine Group.^78^ The vaccine, known as ChAdOx1 nCOV‐19, uses a replication‐deficient ChAd viral vector to encode SARS‐CoV‐2‐S. ChAdOx1 is derived from the ChAd isolate Y25^79^ and has been tested in many pre‐clinical and clinical trials, demonstrating safety with robust humoral and cellular immunogenicity and durable protection.^80^, ^81^, ^82^ At the beginning of the SARS‐CoV‐2 outbreak, the Jenner Institute was conducting CoV vaccine development against MERS‐CoV with collaborative ChAdOx1‐MERS‐CoV phase I clinical trials in Oxford and the Kingdom of Saudi Arabia.^83^, ^84^

Screening, recruitment and vaccination for the ChAdOx1 nCoV‐19 phase I/II clinical trial is currently underway and at the time of writing, approximately 900 healthy volunteers aged 18–55 years have been vaccinated with 5 × 10^10^ viral particles. ChAdOx1 nCoV‐19 or a Meningitis‐ACWY vaccine via intramuscular vaccination, in a randomized 1 : 1 ratio of trial vaccine to control (clinicaltrials.gov: NCT04324606). The primary and co‐primary objectives of this study are to assess the safety and efficacy of the vaccine. The humoral and cellular immune responses to vaccination will be assessed as secondary end points.^85^

The current planned phase II clinical trial will recruit volunteers between the ages of 55 and 70, age 70+ and children age 5–12 years to assess the vaccine across broader demographics. A phase III clinical trial is planned to recruit more than 10 000 volunteers over 18 years old to assess the efficacy of the ChAdOx1 nCoV‐19 vaccine.^86^

## COVID‐19 vaccine development: worldwide clinical trials

In April, CEPI announced the number of COVID‐19 vaccine candidates around the world had exceeded 100.^16^ Here we will focus on those candidates that have entered early clinical trials by April 2020. Table 3 shows data on the protocols for each trial.

**Table 3:** Selected phase I clinical trials for vaccines against SARS‐CoV‐2

Vaccine	Vaccine platform	Location	Cohort size (no. groups)	Dose(s)	Vaccination schedule	Immunological objectives
mRNA‐1273 Moderna Inc. (Cambridge, US)	mRNA in lipid nanoparticle	USA	45 (3)	Group 1: 25 μg RNA Group 2: 100 μg RNA Group 3: 250 μg RNA	Day 1: Prime Day 29: Boost	Antibodies up to 28 days post‐boost
BNT162 BioNtech (Mainz, Germany)	Modified RNA (a1, b1, b2) or self‐amplifying RNA (c2) in a lipid nanoparticle	Germany	200 (4)	Unknown	Day 0 (all groups): Prime Day 21 (a1, b1, b2): Boost	Total and neutralizing antibodies at days 7, 21, 42, 84 and 183 post‐prime
INO‐4800 (Inovio Pharmaceuticals)	DNA	USA	40 (2)	Group 1: 1 mg DNA Group 2: 2 mg DNA	Day 0: Prime Day 28: Boost	Antibodies and cellular response from week 0 to week 28 post‐boost
bacTRL‐Spike Symvivo Corporation (Burnaby, Canada)	DNA with *Bifidobacterium* *longum* delivery vector	Canada	84 (4)	Group 1a: 1 × 10^9^ *Bifidobacterium longum* Group 2a: 3 × 10^9^ *Bifidobacterium longum* Group 3a: 1 × 10^10^ *Bifidobacterium longum* Group 1b, 2b, 3b: Placebo	Day 0: Prime	Antibodies at months 1,3 and 12 post‐prime. Presence of bacTRL‐Spike in stool up to 12 months post‐prime
Ad5‐nCoV ( CanSino Biologics)	Adenovirus viral vector	China	108 (3)	Group 1: 5x10^10^ vp Group 2: 1 × 10^11^ vp Group 3: 1·5 × 10^11^ vp	Day 0: Prime only	Total and neutralizing antibodies and cellular response at days 14 and 28, and months 3 and 6 post‐prime
ChAdOx‐1 nCoV‐19 University of Oxford, AstraZeneca (Cambridge, UK)	Chimpanzee adenovirus viral vector	UK	510 (5)	Group 1a, 2a, 3: 5 × 10^10^ vp Group 1b, 2b: meningitis ACWY standard dose	Day 0: Prime Week 4 (group 3, 10 participants): boost	Total and neutralizing antibodies and cellular response up to 6 months post‐vaccination
COVID‐19/APC Shenzhen Geno‐Immune Medical Institute	Lentivirus‐transduced APC	China	100 (1)	Group 1: 5 × 10^6^ APCs	Day 0: Prime Day 14: Boost Day 28: Boost	Cellular response at days 14 and 28 post‐prime
COVID‐19 Synthetic Minigene Vaccine Shenzhen Geno‐Immune Medical Institute	LV‐DCs and antigen‐specific CTLs	China	100 (1)	Group 1: 5 × 10^6^ LV‐DCs and 1 × 10^8^ CTLs	Day 0: Prime	No immunological outcomes assessed
Inactivated SARS‐CoV‐2 Sinovac Research & Development (Beijing, China)	SARS‐CoV‐2 virus inactivated by β‐propiolactone	China	744 (6)	Group 1 and 4: 600 SU/0·5 ml Group 2 and 5: 1200SU/0·5 ml Group 3 and 6: Placebo	Day 0 (all groups): Prime Day 14 (Groups 1,2 and 3): Boost Day 28 (Groups 4, 5 and 6): Boost	Group 1,2,3: Total and neutralizing antibodies at day 7,14, 21, 28 and 42, cellular response at day 14 and 28 Group 4, 5, 6: Total and neutralizing antibodies at days 28, 35, 42 and 56, cellular response at days 14 and 42

Abbreviations: APC, antigen‐presenting cell; COVID‐19, coronavirus disease 2019; CTL, cytotoxic T lymphocyte; LV‐DC, lentivirus‐transduced dendritic cell; MERS, Middle East respiratory syndrome; SARS‐CoV‐2, severe acute respiratory syndrome coronavirus 2

The table lists: vaccine name, vaccine platform, the trial location, the trial size and number of trial groups, dosing size, vaccination regimen and the primary immunological objective of the trial.

Data sourced from clinicaltrials.gov identifiers: Moderna: NCT04283461, BioNTech: EudraCT 2020‐001038‐36, Inovio: NCT04336410, Symvivo: NCT04334980, CanSino: NCT04313127, University of Oxford: NCT04324606, Shenzhen Geno‐Immune Medical Institute: NCT04299724 & NCT04276896, Sinovac Biotech: NCT04352608.

### mRNA platforms

#### mRNA1273 (Moderna) and BNT162 (BioNTech)

The Moderna and BioNTech platforms are messenger RNA (mRNA) molecules expressing SARS‐CoV‐2‐S, contained within lipid nanoparticles to facilitate entry of mRNA into the host cells.^87^, ^88^ Once inside the host cell, the S protein will be expressed and induce antibody responses. The Moderna vaccine was the first to progress to phase I clinical trials in humans.^89^

The Moderna platform has been used in phase I clinical trials for several pandemic potential diseases, including MERS, Zika (clinicaltrials.gov NCT04064905) and pandemic influenza.^87^ Phase I data for a pandemic influenza vaccine focused on a cohort vaccinated with 25, 50 or 100 µg of vaccine. The vaccine had a good safety profile and showed seroconversion in volunteers who received the highest dose. Crucially, 87% of volunteers who seroconverted developed neutralizing antibodies. The platform did not however generate any measurable cell‐mediated cytokine responses.^87^

The BioNTech platform has been used in Zika virus vaccine development. Vaccination with a 30‐µg or 50‐µg dose in C57BL/6 mice and rhesus macaques respectively induced neutralizing antibody titres against Zika virus, and protected challenged animals from detectable viraemia.^88^ This mRNA platform is also being used in phase I/II clinical trials for several cancer vaccines.^90^

### DNA vaccines

#### INO‐4800 (Inovio Pharmaceuticals) and bacTRL‐Spike (Symvivo Corporation)

INO‐4800, and the previous Inovio vaccine INO‐4700, express either SARS‐CoV‐2‐S or MERS‐CoV‐S respectively within an identical DNA vaccine vector.^91^ The vaccine is administered through intramuscular injection, followed by electroporation of the injection site. The need for electroporation may limit the ability of INO‐4800 to be extended to the scales needed for a global pandemic and may be difficult to administer worldwide.

INO‐4700 showed promising immunogenicity in a phase I clinical trial after multiple immunizations.^92^ The trial administered three doses of vaccine, and was split across high, medium and low groups. Initial seroconversion peaked at 86% of the total cohort before falling slightly by the trial end point. Titres of neutralizing antibodies showed similar patterns, peaking at 43% of the cohort 2 weeks after final vaccination, before being detected in only 3% of the cohort at the trial end point. Seventy‐six per cent of the total cohort also showed an interferon‐*γ* T‐cell response against MERS‐specific peptides.^92^

Pre‐clinical animal models of INO‐4800 have been published in pre‐print. These demonstrate seroconversion in all animals and robust T‐cell interferon‐*γ* responses 10 days post‐vaccination against peptides spanning SARS‐CoV‐2‐S (pre‐print ahead of publication, https://doi.org/10.21203/rs.3.rs‐16261/v1).

The bacTRL platform from Symvivo Corporation uses engineered probiotic *Bifidobacterium longum* to deliver a DNA vaccine expressing SARS‐CoV‐2‐S into intestinal cells. The phase I trial of a COVID‐19 vaccine will also be the first‐in‐man study of the bacTRL platform, so no previous immunological data are available (clinicaltrials.gov NCT04334980).

### Viral vectored vaccines

#### Ad5‐nCoV (CanSino Biologics), APCs and LV‐DC/CTLs (Shenzhen Geno‐Immune Medical Institute)

The Ad5 platform from CanSino Biologics demonstrated safety in phase I/II trials for Ad5‐EBOV,^54^ a vaccine against the Zaire strain of EBOV. In addition, Ad5‐EBOV induced humoral immune responses, with 100% seroconversion of vaccines in a phase I trial. Volunteers also exhibited an interferon‐*γ* T‐cell response, significantly different from the placebo group, suggesting the induction of limited cell‐mediated immunity.^54^ This vaccine has subsequently been licenced for emergency use in China against EBOV.

In the phase I Ad5‐EBOV trial, the study found that participants with pre‐existing Ad5 neutralizing antibodies showed significantly lower humoral and cellular responses to the EBOV glycoprotein than participants that were seronegative against Ad5.^54^

The Shenzhen Geno‐Immune institute are using lentiviruses to transduce dendritic cells (DCs) and antigen‐presenting cells (APCs) to induce cytotoxic T‐cell responses in individuals who have developed COVID‐19. Dendritic cells are a subset of APCs that can be modified to express and present antigens, and this property has been used in phase I trials with tumour‐associated and neo‐antigen cancer vaccines.^93^ The trials will test both the efficacy of APCs as a vaccine alone (clinicaltrials.gov NCT04299724), and administration of modified DCs with donor cytotoxic T cells as a combination therapeutic and vaccination (clinicaltrials.gov NCT04276896).

### Inactivated vaccines

#### Inactivated SARS‐CoV‐2 (Sinovac Research and Development)

Sinovac has published pre‐clinical data on the efficacy of an inactivated SARS‐CoV‐2 vaccine in a macaque challenge model.^40^ Rhesus macaques received three vaccine doses of either 3 or 6 µg at 1‐week intervals. Titres of IgG and neutralizing antibody after the third vaccination were similar to those induced by natural infection in recovered patients. T‐cell responses were not reported. Macaques were challenged 8 days after the third vaccination and displayed a reduction in viral load compared with unvaccinated animals; encouragingly, there was no evidence of ADE or Th2‐skewed immune responses in vaccinated animals.^40^

## COVID‐19 vaccine directions

The COVID‐19 pandemic has driven vaccine research and development into unprecedented territory. Non‐clinical suppression strategies involving contact tracing and social distancing have been employed globally to varying degrees.^17^ There is growing evidence that these interventions have had considerable impact on ‘flattening the curve’ of the epidemic, thus reducing the burden on overstretched health‐care systems, and allowing time for vaccine and antiviral development. However, relaxing social distancing measures too soon may result in a second peak in infections.^94^ The social, economic and health effects of these measures on society will be fully realized over the coming months, although it is likely that the cost of these strategies will be steep. Furthermore, healthcare facilities have been subjected to intense pressure and high demand, with supplies of personal protective equipment in short supply across the UK and internationally.^95^ Together, these limitations put the lives of health‐care workers and patients at higher risk.^96^ The length of natural protection post‐exposure is currently unknown, and could result in regular circulation of SARS‐CoV‐2 if immunity is not long‐lived.^97^ The development of a safe and effective vaccine, therefore, is vital for mass protection of those most at risk from COVID‐19‐induced disease. This will reduce the number of hospitalized cases, subsequently relieving the burden on health‐care systems, and will allow for relaxation of physical distancing interventions.

### What is needed from a vaccine for COVID‐19?

The ideal candidate COVID‐19 vaccine would have good safety and immunogenicity profiles in all age groups and demographics including pregnant women and immunocompromised individuals, and would generate robust cellular and humoral immunity with a single vaccination, which could potentially be boosted for long‐lasting memory durability.^52^, ^63^ Single‐shot efficacy was demonstrated in clinical trials during the latter half of the 2013–2016 EBOV outbreak, where one vaccination of rVSV‐ZEBOV conferred up to 100% protection for at least 84 days.^55^ A ChAd vectored vaccine (ChAd3 EBOZ GP) induced similar immunogenicity, also with a single dose, suggesting that multiple viral vector vaccines are effective at inducing high levels of immunity after a single dose.^98^ Single‐dose protective efficacy would offer fast protection to frontline health‐care workers and those in close contact with infected individuals, with additional booster vaccines to extend duration of immunogenicity if needed.^97^ Public concerns surrounding vaccine safety may be heightened during the outbreak of an unknown pathogen and unclear scientific reporting. Vaccine hesitancy because of perceived risk is a globally observed phenomenon,^99^ and care must be taken to ensure that the public is aware that full safety and regulatory requirements of a new and rapidly developed vaccine against SARS CoV‐2 have been completed with due care.

The ability to generate large quantities of GMP vaccine in a short duration of time is also essential. The cost of producing COVID‐19 vaccines for public use will be steep and will exceed CEPI’s $2 billion fundraising goal, which will establish GMP manufacturing sites, but not cover eventual vaccine manufacturing.^17^ Global input will be necessary to fund and produce vaccines at multiple sites across the world, while ensuring fair and equitable distribution. It is hoped that more than one vaccine candidate undergoing current development and clinical testing will be suitable for mass vaccination in the coming year. In this manner, global need will hopefully be adequately addressed.

Unparalleled vaccine research and development is ongoing for the SARS‐CoV‐2 epidemic. An efficacious and publicly available vaccine will significantly reduce the impact of the current and possible future epidemic peaks, so reducing the burden on national health‐care systems. It is vital, however, that we continue to develop tenable vaccines that are both safe and immunogenic, that can be manufactured at scale and that can be distributed to both economically stable countries and to low‐ and middle‐income countries, ensuring equal access.

## Disclosures

Teresa Lambe is named as an inventor on a patent application covering a SARS‐CoV‐2 (nCoV‐19) vaccine. The remaining authors declare no competing interests.
